# Impact of surgery on rehabilitation care and quality of life perceived by patient with post-stroke upper limb spasticity: Study protocol for a randomized controlled trial

**DOI:** 10.1371/journal.pone.0322588

**Published:** 2025-04-30

**Authors:** Patricia Hurtado-Olmo, Ángela González-Santos, Laura del Olmo Iruela, Belén Castro-Ropero, Lourdes Zúñiga-Gómez, Ana Isabel Bueno-García, Pilar Guijosa-Campos, Basilio Gómez-Pozo, Fidel Hita-Contreras, Pedro Hernández-Cortés

**Affiliations:** 1 Upper Limb Surgery Unit, Orthopedic Surgery Department, San Cecilio University Hospital of Granada, Spain; 2 Department of Physical Therapy, Faculty of Health Science, University of Granada, Granada, Spain; 3 BIO277 Group, A02-Cuídate, Instituto de investigación biosanitaria Ibs. Granada, Spain; 4 Rehabilitation Department, San Cecilio University Hospital of Granada, Granada, Spain; 5 Unidad Clínica de Prevención, promoción y vigilancia de la salud. Distrito Sanitario Granada SAS, Granada, Spain; 6 Department of Health Sciences, Faculty of Health Sciences, University of Jaén, Jaén, Spain; 7 Surgery Department, School of Medicine, Granada University, Granada, Spain; 8 IBS. Instituto Biosanitario de Granada, Granada, Spain; Public Library of Science, UNITED KINGDOM OF GREAT BRITAIN AND NORTHERN IRELAND

## Abstract

**Background:**

Stroke is the principal cause of permanent disability in adult age, and many patients require lifelong medical treatment and care from others for their daily activities. It has enormous repercussions on the work and social lives of patients and their families and involves major economic expenditure. Post-stroke spastic upper limb is usually treated with rehabilitation, occupational therapy, and periodical injections of botulinum toxin, while surgical correction is now seldom considered. However, there has been no clinical trial to compare between surgical and toxin treatments. The primary aim of this study is to compare outcomes between surgery and a conventional approach with botulinum toxin in patients with post-stroke upper limb spasticity.

**Methods:**

A two-arm (surgical treatment [n = 22] *vs*. botulinum toxin [n = 22]) randomized clinical trial (RCT) will be performed to compare the efficacy of surgery with that of botulinum toxin treatment in patients with post-stroke upper limb spasticity. Data will be collected at baseline and at 6 and 12 months of follow-up on functionality, hygienic status, quality of life, sleep quality, anxiety/depression levels, and functional magnetic resonance imaging (fMRI)-measured brain activity. Healthcare and care costs will be compared between the groups.

**Discussion:**

This research is set in the context of chronic diseases, aging, and functional/mobility limitations. The results can be expected to have a major impact, because the high prevalence of stroke and the severe associated disability means that an enormous number of patients can benefit from improved treatment protocols, and a more rational use of resources would yield considerable economic benefits for health and care systems. Our expectation is that outcomes would be more favorable with surgery. However, the aim is not to exclude any approach but rather to explore how the potential and indications of each treatment could be integrated within a multidisciplinary therapeutic protocol in a complementary manner.

**Trial Registration**: ClinicalTrials.gov (NCT06392633). Registered on 30 April 2024.

## Introduction

Stroke is associated with high mortality and morbidity rates and represents a public health problem of the first order. It is the main cause of permanent disability in adult age [1] and responsible for spasticity in 38% of patients, who require chronic medical treatment and depend on others for their daily living activities [2], with a severely impaired quality of life [3].

Population aging and improved stroke survival rates have raised the number of individuals with stroke sequelae needing specialist care, rehabilitation, and support, increasing the burden on families, society, and health systems [4]. Although there have been improvements in the management of acute stroke episodes, less attention has been paid to the chronic care of sequelae, leading many patients to feel abandoned after their discharge from hospital, with the need to manage and defray their care needs themselves due to inadequate support from the public healthcare system. Caregivers play a key role in the care of stroke survivors, and it is important to capture and predict changes in caregiver quality of life and burden for the design of tailored interventions [5].

Various surgical techniques are applied to optimize the function, reduce the pain, and improve the hygiene and esthetics of the spastic upper limb [6]. Corrections can be performed by single-event multilevel surgery, with a combination of soft tissue releases and elongations, tendon transfers, and joint stabilization procedures [7], or by focusing on the nerve as spasticity vehicle and using hyponeurotization or hyperselective neurectomy [8] or even rhizotomy of the C7 root with contralateral transfer [9–11].

Currently, the first-choice treatment of localized spasticity is intramuscular injection of botulinum toxin A [12,13]. This has generally replaced surgery, which is now seldom considered and may be under-prescribed [14,15], although no studies have compared these modalities.

Surgery for upper limb spasticity has been associated with high satisfaction levels in patients and caregivers and with low complication rates [16]. Although it cannot be performed in many patients, good long-term outcomes have been reported for the surgical treatment of non-functional or hygienic upper limbs, [7,17]. However, scientific evidence of its effectiveness remains weak, generally based on retrospective case series with no control group or on heterogeneous study populations of patients with stroke, child cerebral palsy (CCP), or post-traumatic brain injury (TBI), among others.

The success of therapeutic procedures has usually been evaluated in terms of outcomes from the perspective of the physician. Less attention has been paid to the impact of the disease on daily life, social functioning, and patient satisfaction or to the predicted utilization of health resources. However, increasing consideration is being given to the evaluation of outcomes from the perspective of patients [18], including quantification of their own perception of their health and the impact of symptoms or treatments on health-related quality of life (HRQOL). HRQOL is a multidimensional concept that combines physical, mental, emotional, and social functioning with wellbeing [19]. Information from patients can be useful for the design and implementation of interventions by health teams, for comparison of the effectiveness of different treatments (e.g., medical *vs*. surgical), and for the modification of surgical techniques, among others.

Some studies have assessed the impact of therapy with rehabilitation and botulinum toxin on the quality of life of patients with stroke [20,21], but only Van Heest et al. [22] have examined the effects of a surgical approach on quality of life, and their study population comprised pediatric patients with CCP, whose profile differs from that of stroke patients.

The application of functional MRI (fMRI) in patients with stroke sequelae allows the recovery of motor functions to be monitored and brain activity to be compared with that of healthy controls [23], correlating post-treatment findings with improvements in social ability, expression, and cognitive capacity [24].

Besides the peripheral effects of botulinum toxin treatment, it has been associated with brain modulation in fMRI studies of patients with stroke [25,26] or TBI [27]. However, fMRI has not been used to investigate changes that may result from spastic hand surgery, except in the trial by Zheng et al. on contralateral C7 nerve root transfer [11].

Randomized clinical trials (RCTs) are required to compare outcomes, economic cost, and impact on quality of life between the surgical and botulinum toxin treatment of patients with post-stroke upper limb spasticity.

## Materials and methods

This protocol follows the Standard Protocol Items by Recommendations for Interventional Trials (SPIRIT) checklist and diagram (Table 1).

**Table 1:** Details of enrollment, interventions and assessments following the SPIRIT diagram. MRI: Magnetic Resonance Imaging.

	STUDY PERIOD				
	Enrolment	Allocation	Post-allocation		Close-out
TIME POINT	0-90 days before	0	Baseline	6 months	12 months
ENROLMENT					
Eligibility Screening	X				
Informed consent		X			
Initial evaluation	X				
Allocation		X			
INTERVENTIONS					
Surgery					
Botulinum Toxin					
ASSESSMENT					
***Baseline***					
Clinical and sociodemographic data			X		
Functionality evaluation			X	X	X
Caregiver burden evaluation			X	X	X
Quality of life evaluation			X	X	X
Sleep, anxiety, and depression assessments			X	X	X
Structural and functional MRI			X	X	X
*Outcome variables*					
Changes in functionality				X	X
Changes in caregiver burden				X	X
Changes in quality of life				X	X
Changes in sleep quality, anxiety, and depression				X	X
Structural changes (gray and white matter) on MRI				X	X
Functional changes on MRI				X	X

### Aims, design, and setting

The primary aim of this study is to compare outcomes between surgery and a conventional approach with botulinum toxin in patients with post-stroke upper limb spasticity.

The secondary aims are:

1. To compare functional and hygienic changes (non-functional: esthetics, pain, and ease of care) in the spastic upper limb between surgery and botulinum toxin therapy using the House [28], Fugl Meyer [29], GAS [30], Zancolli [31], Modified Ashworth-Bohanon [32], Keenan [33] and the pain Visual Analog Scale (VAS) [34]
2. To compare the impact on quality of life related to care and psychosocial risks of surgery *versus* botulinum toxin therapy in patients with post-stroke upper limb spasticity using SF 36 [35] and the Newcastle stroke-specific quality of life measure [36]
3. To compare the effects on sleep quality (PSQI) [37], anxiety and depression (HADS) [38] of surgery *versus* botulinum toxin therapy in patients with post-stroke upper limb spasticity.
4. To compare the effects on caregiver burden of surgery *versus* botulinum toxin therapy using the Zarit [39] and Caregiver Burden scales [7].
5. To compare the effects on brain structure and function of surgery *versus* botulinum toxin therapy using fMRI.
6. To perform a comparative cost-effectiveness analysis between patients with stroke sequelae treated with surgery *versus* botulinum toxin therapy.

The study will be a two-arm RCT in patients with post-stroke upper limb spasticity, randomly assigning participants to a Control group for botulinum toxin treatment or an Experimental Group for surgery (1:1 allocation ratio).

It will be conducted in Granada (940,974 inhabitants) and Jaén (620,242 inhabitants), two cities in in southern Spain, and will be led by Clínico San Cecilio University Hospital (CSCUH), Ciudad de Jaén University Hospital (CJUH), and the Surgery Department of Granada University.

The Project for this trial (Ref: PI20/01574) is funded by the *Instituto de Salud Carlos III* (ISCIII) and co-funded by the European Union. This research is part of the doctoral thesis of Patricia Hurtado-Olmo, developed under the Doctoral Program in Clinical Medicine and Public Health at the University of Granada (Spain).

A sample of 44 adults with post-stroke spastic upper limbs will be recruited for the study between 1^st^ January 2023 and 30^th^ June 2025.

### Participants

**Fig 1:**
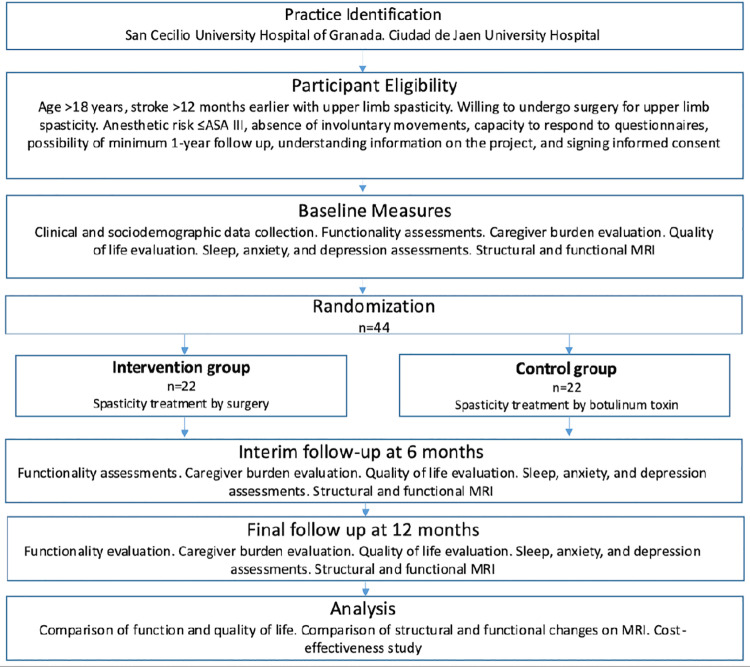
Overview of the study.

Participants will be recruited according to the following inclusion criteria: a) age > 18 years, b) presence of post-stroke upper limb spasticity, c) minimum time interval of 12 months since the stroke, d) agreement to surgery for upper limb spasticity and inclusion on the hospital surgical waiting list, and e) written informed consent to participation in the study (by the patient or legal representative). Exclusion criteria will be anesthetic risk (ASA) ≥IV, presence of involuntary (extrapyramidal) movements, inability to adequately respond to questionnaires, the presence of deformities not treatable by surgery, and the impossibility of follow-up at a minimum of 12 months. (Fig 1).

Any patient who starts the study can abandon it at any time due to health, administrative or travel problems.

The sample size has been estimated for 5% type I error probability, 80% power, and a large effect size difference (0.8 standard deviations). Balanced one-way analysis of variance power calculation yielded K = 2, n = 16.71472, and f = 0.5. Accordingly, 34 patients (17 per group) are required with a minimum follow-up of 12 months. The sample size is set at 44 patients (22 patients in each group) to cover possible losses to the follow-up of 20%.

### Ethics approval and consent to participate

This study has received approval from the clinical research ethics committee of the Junta de Andalusia (Spain) on 7 December 2020 (code: 6hWMS793PFIRMAj6fT0fx7yTDkZ6SF). Authors have intentions to obtain informed consent to participate in the study from participants (both documents are sent as additional files). Patient participation will be entirely voluntary. They will sign the corresponding informed consent in accordance with ethical criteria in the 2013 modification of the Helsinki Declaration for the performance of research projects and with national legislation on clinical trials (Law 29/2006 26 July, modified in July 2013). The confidentiality of study material will comply with national legislation on personal data protection and digital rights (Law 3/2018, 5 December).

### Recruitment

PHO will prepare a list of patients with stroke sequelae for > 1 year from the records of the Rehabilitation Departments of the Granada and Jaen University Hospitals (Andalusia, Spain) and from associations of patients with acquired brain damage (ADACEA [Jaen], NEUROAFEIC, and AGREDACE [Granada]). A Rehabilitator (LZG, LOI, BCR) on the research team will contact candidate participants to explain the study, confirm eligibility, and obtain informed consent.

At enrolment, a clinical assessment of all participants will be performed by a Rehabilitator at the CSCUH to verify the diagnosis and confirm that eligibility criteria are met. The clinical assessment consists of a neurological examination and a short, standardized medical history interview. Baseline data will be collected at this session.

Before randomization, all selected patients will be examined by a hand and upper limb surgeon (PHC) for assessments of the shoulder, elbow, wrist, and hand, using peripheral nerve blockade anesthetics when required. Patients will receive individualized proposals for single-event surgery with a combination of soft tissue procedures (tenotomy, tendon lengthening, or tendon transfer), selective neurectomies, and bone procedures (osteotomy and arthrodesis).

### Randomization

Randomization of patients will be conducted by an independent member of the research team (BGP) not involved in the data collection. The allocation sequence will be based on a computer-generated random number. Randomization will be conducted after the recruitment of individual participants is completed to reduce the risk of selection bias.

### Intervention group

Patients assigned to the surgery group will be placed on the HUCSC waiting list and will undergo anesthetic risk assessment by the Anesthesiology Department. Antiaggregant or anticoagulant therapy will be perioperatively replaced with low-molecular weight heparin (LMWH).

Patients will be hospitalized the day before surgery and anesthetized in accordance with the preanesthetic assessment. The proposed treatment will consist in single-event multilevel surgery that includes the shoulder (tenotomy or pectoralis major and/or subscapularis lengthening), elbow (brachial biceps and anterior brachialis lengthening or musculocutaneous nerve hyponeurotization), forearm (pronator teres tenotomy or Scaglietti procedure [flexor-pronator mass release]), wrist (arthrodesis, tendon lengthening, tendon transfers, particularly superficialis-to-profundus tendon transfers), and hand (tendon lengthening, neurotomies of the motor branch of the ulnar nerve, and arthrodesis).

Hospital discharge is expected to be possible after drainages are removed and the patient has an adequate degree of analgesia. Patients will frequently undergo plaster cast immobilization and will be referred to the rehabilitation department for physical therapy after cast removal. Botulinum toxin will not be administered during the postoperative period.

### Control group

Patients in the control group will be treated with botulinum toxin in the rehabilitation department, receiving injections in upper limb spastic muscles at one session every 4 months.

### Data collection

Evaluations will be conducted by a specialist physician with >25 years’ experience at three time points: at baseline (t0) and at 6 months (t1) and 12 months (t2) post-intervention. Evaluations will be carried out in single sessions at Granada University School of Medicine except for the fMRI scan, which will be performed at the Mind, Brain, and Behavior Research Center of the University of Granada by technical staff at this center. (Fig 1).

Data will be collected on the following clinical and sociodemographic variables: age, sex, type of stroke, date of stroke, interval between stroke and treatment, gait capacity on the Functional Ambulation Classification (FAC) scale [40], upper limb lesion (functional or hygienic), cognitive status [41], family support, need for assistance (Barthel index) [42], and anesthetic risk (ASA) [43], classifying upper limb lesions as functional or hygienic hand and categorizing elbow, wrist, and hand deformities according to Keenan et al [33].

#### Functionality evaluation (main variable).

This evaluation will be performed at each time point (0, 6, and 12 months) using the following instruments:

1. Modified Ashworth spasticity scale [32], which measures the degree of spasticity from 0 to 5. Significant improvement at one or more of the five evaluated sites (elbow, forearm, wrist, thumb, fingers) will be considered a positive outcome.
2. Hand function scale by House et al. [28], which considers nine functional grades.
3. Zancolli hand classification [31].
4. Fugl-Meyer scale for post-stroke recovery. It includes 33 items scored from 0 to 2 and a scale for shoulder-wrist and wrist-hand domains scored from 0 to 66 [29].
5. VAS, which provides a subjective estimate of pain, using a line ranging from 0 “no pain” to 10 “worst pain imaginable” [34].

#### Evaluation of hygienic or nonfunctional achievements (secondary variable) and caregiver burden.

This evaluation will be performed at each time point (0, 6, and 12 months) using the following instruments:

1. “GAS score” [30], a 5-point scale score for hygiene, esthetics, and pain domains.
2. Caregiver Burden Scale [7], which rates the degree of difficulty of caregivers in four basic activities (nail-cutting, palm washing, axilla washing, upper limb dressing) using a 5-point “Likert” scale.
3. Zarit Caregiver Burden questionnaire [39].

#### Quality of life evaluation.

This evaluation will be performed at each time point (0, 6, and 12 months) using a generic questionnaire (Spanish-validated version of SF-36 [35]) and a quality-of-life-specific questionary (New Castle Stroke-Specific Quality of Life measure [36]).

#### Evaluation of sleep quality, anxiety, and depression.

This evaluation will be performed at each time point (0, 6, and 12 months) using the following instruments:

Pittsburgh Sleep Quality Index (PSQI) questionnaire (Spanish version validated by a member of the present research team [37]), which includes 19 self-evaluated questions covering seven domains: quality, latency, duration, habitual efficiency, discomfort, use of sleeping medication, and day-time dysfunction. Item and domain scores range from 0 to 3 (higher score = worse quality).

Hospital Anxiety and Depression Scale (HADS) questionnaire (validated Spanish version [38]), which includes 14 items with scores from 0 to 3 (higher score = more severe symptom).

#### Evaluation of brain activity, structure, and function.

This evaluation will be performed at each time point (0, 6, and 12 months) by using the fMRI at rest and with repeated attempts of patients at the flexion-extension of elbow, wrist, and fingers. Anatomical and functional volumes will be obtained from MRI and fMRI images using a 3T Siemens Prisma fMRI. A three-dimensional volume will be acquired in each patient by using a T1-weighted 3D-turbo-gradient-echo (3D-TFE) sequence in sagittal orientation, with 1x1x1 mm resolution (160 slides, FOV = 240 mm2, 256x256x160 matrix, 8 s repetition time, 4 ms echo time, 8º flip angle, and 191 Hz/pixel band width). Each functional examination will include 180 volumes with a T2*-weighted echoplanar imaging (EPI) cranium sequence using the following parameters: repetition time = 2.0 s, echo time = 30 ms, flip angle = 70º, 33 slices with 3-mm width and 1-mm gap, 3 mm in-plane resolution). MRI images will be analyzed using SPM (version 8, http://www.fil.ion.ucl.ac.uk/spm/software/spm8/) and FSL (version 5.0, http://fsl.fmrib.ox.ac.uk/fsl/fslwiki/) open software packages. In anatomical images, the DARTEL algorithm (Diffeomorphic anatomical registration through exponentiated Lie algebra) will be used to measure grey matter and white matter volumes. Subcortical structures (hippocampus, basal ganglia, amygdala, nucleus accumbens, and brainstem) will be segmented using the FIRST algorithm implemented in FSL, which allows the volume and shape of these structures to be determined. The shape measurement will use the medial distance from each point of the structure surface to its medial axis. The general linear model implemented in the SPM8 and FSL packages will be used for the statistical analysis. The random field theory (RFT) correction will be applied to address the problem of multiple comparisons, and SPM12 (http://www.fil.ion.ucl.ac.uk/spm/software/spm12/) will be used for fMRI image processing and analysis.

#### Evaluation of health costs.

Health resources consumed by participants will be calculated in accordance with an economic model developed to compare surgical treatment with botulinum toxin alone in the Spanish setting. Clinical records will be reviewed to gather data on medication, specialist visits, primary health care consultations, and health transportation. Prices in the Pharmaceutical School Council Catalog will be applied to medications. Information in the Spanish eSalud health cost database will be used to generate a unitary cost for primary and specialized care consultations, emergencies (hospital and primary care), and health transportation. Hospitalization costs will be based on mean values published by the Ministry of Health, Social Policy, and Equality for the corresponding diagnostic groups. Account will also be taken of the need for occupational therapy. Costs of the main caregiver and secondary caregivers will be based on the hours they devote to care activities, applying the market hourly rate for similar services. In addition, a cost-utility analysis will be performed based on patient-reported quality-of-life results.

Outcome measures are summarized in Table 2.

**Table 2:** Summary of outcome measures. GAS: Goal Attainment Scale; HADS: Hospital Anxiety and Depression Scale; MRI: Magnetic Resonance Imaging; PSQI: Pittsburgh Sleep Quality Index; SF-36: 36-item Short Form Survey.

Outcomes	Time point	Measurement definition
Change in functional and hygienic status of the affected upper limb	6 and 12 months	Measured using House, Keenan, Zancolli, Fugl Meyer, GAS, Asworth and VAS scales
Change in quality of life	6 and 12 months	Measured using SF 36 and New Castle Stroke-Specific quality of life scale
Changes in sleep quality	6 and 12 months	Measured using the PSQI
Changes in anxiety and depression	6 and 12 months	Measured using HADS score
Changes in caregiver burden	6 and 12 months	Measured using Carer Burden and Zarit scales
Changes in brain structure	6 and 12 months	Measured using MRI
Changes in brain function	6 and 12 months	Measured using fMRI
Treatment costs	12 months	Using model to simulate costs and quality-adjusted life years. Unit costs from published Spanish sources

PGC and BGP will be the data monitoring committee; who will maintain continuous contact with patients to reduce the risk of dropout and will ensure that no patient misses any assessment during the follow-up period, and will be responsible for reporting any adverse event related or not to the treatment applied, as well as making the decision to terminate the trial. Auditing trial conduct will be carried out monthly.

Access to the final trial dataset will be allowed to principal investigator, data monitoring committee, and responsible biostatistician.

### Data analysis

The Student’s t-test will be used for between-group comparisons of continuous variables, applying the Mann-Whitney U test when the distribution is non-normal. Repeated-measures regression analysis will be conducted for within-group comparisons between follow-up visits and baseline. McNemar’s Chi-square test or Fisher’s exact test will be used for between-group comparisons of discrete variables (e.g., Ashworth spasticity scale or Caregiver Burden Scale) and McNemar’s Chi-square test for within-group comparisons. Pearson’s correlation coefficient analysis will be applied to evaluate relationships between functional scores and the results of health, anxiety-depression, and sleep quality questionnaires. Multivariate linear regression will be performed to determine whether the functional improvement behaves as an independent variable with significant influence on aspects of psychosocial quality of life.

The fMRI results will be expressed descriptively and submitted to correlation analysis. The corrected BOLD response associated with the upper limb movement will be analyzed using a general linear model in SPM8, considering the aforementioned confounders and independent variables. The regression coefficients obtained for each patient will be analyzed by ANOVA to identify the brain regions that manifest effects associated with the variables of interest. Multiple comparison correction criteria will be applied for statistical decisions, resulting in an alpha error rate ≤0.05 throughout the trial.

## Discussion

This article presents a two-arm RCT designed to compare the outcomes of surgery for upper limb spasticity with those of botulinum toxin therapy in patients with post-stroke spastic sequelae. We shall compare the effects of functional and hygienic changes on quality of life, sleep quality, anxiety, depression, and fMRI-measured brain structure and activity at baseline and at 6 and 12 months of follow up. We shall also evaluate the health and care costs associated with each approach.

Botulinum toxin is currently the first-line choice for regional post-stroke spasticity of the upper limb [44]. However, surgery generally obtains good long-term outcomes, with a low rate of major complications, and there is a lack of comparative data on the effectiveness, safety, and economic cost of the two approaches. In a study of patients with stroke or TBI from the National Inpatient Sample database (2001–2012) treated with upper limb reconstructive surgery, Beutel et al. [45] reported that 80% of 730,000 new cases of stroke/year survived the acute episode, 76% of survivors developed spasticity, and 50% of spastic patients could benefit from surgery [46]. However, surgery was performed in only 2,132 patients over the 12-year study period, i.e., < 1% of suitable candidates for surgery, indicating that upper limb reconstructive surgery may be considerably under-prescribed in this patient population.

To our best knowledge, no clinical trial has been published that compares the efficacy of surgery with that of botulinum toxin therapy.

In 2023, our group published a systematic review on the surgical treatment of spastic upper limb [47]. We concluded that surgery is a useful, safe, and long-lasting option for post-stroke spastic upper limbs, although most patients in the review were candidates for hygienic improvements alone. However, the design and heterogeneous patient populations of the reviewed studies yielded only a low level of evidence. We also called for clinical trials to compare between surgery and botulinum toxin in the treatment of these patients.

We expect that surgery will achieve improvements in all study parameters. The aim is not to exclude either approach but rather to explore how their potential and indications might be integrated within a multidisciplinary treatment protocol in a complementary manner.

This research is set in the context of chronic diseases, aging, and functional/mobility limitations. The results can be expected to have a major impact, because the high prevalence of stroke and the severe associated disability mean that an enormous number of patients can benefit from improved treatment protocols, and a more rational use of resources would yield significant economic benefits for health and care systems.

We anticipate that the findings of this RCT will contribute to our understanding of the role of upper limb surgery for spasticity in combination with conventional therapy. They will also provide evidence on the specific target group of patients who could gain most from surgery and on the safest and most appropriate procedures to consider. Future results will be communicated through articles in high-impact journals and international conferences on traumatology, rehabilitation and neurology.

The RCT design is a major strength of this study. On the other hand, it will be limited to a specific geographical area and to the public health sector, which may hinder the extrapolation of results to other populations. Other expected limitations include the heterogeneous nature of stroke patients in terms of their baseline injury and upper limb sequelae. In addition, the surgery will not be a single procedure but will involve different steps according to the needs of the patient, making analysis more complex. Multivariate analysis of the applied techniques may be required. The quality of life and psychosocial repercussions are not the only variables that could be selected, and functional and quality of life outcomes are influenced by environmental conditions and patient characteristics such as tiredness, cognitive level, and expression ability. Exclusion criteria have been selected to minimize these influences. Economic analyses may also over- or under-estimate costs, and it can be highly challenging to quantify all informal, secondary, or indirect costs. Nevertheless, information on the economic impact of diseases and their treatments is crucial for health policy planning and evaluation.

## Trial status

This paper is based on protocol version 2, dated 10 December 2022. Recruitment to the study began on 9 January 2023. Approximate date of recruitment completion: June 2025. The trial is registered on Clinical Trials.gov with the registration number NCT06392633.

## Abbreviations

ISCIII: Instituto de Salud Carlos III
RCT: Randomized Clinical Trial
fMRI: Functional Magnetic Resonance Imaging
CCP: Child Cerebral Palsy
TBI: Traumatic Brain Injury
HRQOL: Health- Related Quality of Life
VAS: Pain Visual Analogue Scale
PSQI: Pittsburgh Sleep Quality Index
HADS: Hospital Anxiety and Depression Scale
CSCUH: Clínico San Cecilio University Hospital
CJUH: Ciudad de Jaén University Hospital
ASA: Anesthetic Risk Scale
LMWH: Low-molecular weight heparin
t0: Baseline
t1: 6 months follow- up
t2: 12 months follow- up
FAC: Functional Ambulation Classification
